# In Vitro and In Vivo Study of the Short-Term Vasomotor Response during Epileptic Seizures

**DOI:** 10.3390/brainsci10120942

**Published:** 2020-12-07

**Authors:** Anna Volnova, Vassiliy Tsytsarev, Maria Ptukha, Mikhail Inyushin

**Affiliations:** 1 Biological Faculty, Saint Petersburg State University, St Petersburg 199034, Russia; 2 Institute of Translational Biomedicine, Saint Petersburg State University, St Petersburg 199034, Russia; ptukhamaria@yandex.ru; 3 School of Medicine, University of Maryland, Baltimore, MD 21201, USA; tsytsarev@umaryland.edu; 4 Department of Physiology, School of Medicine, Universidad Central del Caribe, Bayamon, PR 00956, USA

**Keywords:** astrocytes, cerebral blood circulation, epilepsy, epileptic seizures, vasoconstriction, vasodilation, vasomotor activity, 4-aminopyridine

## Abstract

Epilepsy remains one of the most common brain disorders, and the different types of epilepsy encompass a wide variety of physiological manifestations. Clinical and preclinical findings indicate that cerebral blood flow is usually focally increased at seizure onset, shortly after the beginning of ictal events. Nevertheless, many questions remain about the relationship between vasomotor changes in the epileptic foci and the epileptic behavior of neurons and astrocytes. To study this relationship, we performed a series of in vitro and in vivo experiments using the 4-aminopyridine model of epileptic seizures. It was found that in vitro pathological synchronization of neurons and the depolarization of astrocytes is accompanied by rapid short-term vasoconstriction, while in vivo vasodilation during the seizure prevails. We suggest that vasomotor activity during epileptic seizures is a correlate of the complex, self-sustained response that includes neuronal and astrocytic oscillations, and that underlies the clinical presentation of epilepsy.

## 1. Introduction

About 2% of the population experiences an unprovoked epileptic seizure at least once in their lives, and epilepsy research has a long history of in vitro and in vivo experimentation [1]. These events are clearly recognizable on electroencephalogram recordings or by psychosomatic manifestation, but the underlying biological mechanisms are not yet fully understood [2,3,4]. Human epilepsy is most often defined as a manifestation of periodic self-sustaining paroxysmal dysfunction of the brain, characterized by excessive synchronized firing of neurons united in a common network. Unlike normal neurovascular coupling, epileptic seizures place supranormal demands on the brain’s regulatory mechanisms as a result of a pathological increase in the rate of oxygen consumption following both local and ictal events [4]. An early hypothesis proposed that neuronal damage following severe epileptic seizures was caused by local cerebral hypoxia, but this theory was refuted by later studies [5,6,7]. Epileptic seizures include dynamic changes of intracellular and extracellular ionic concentrations as well as changes in the processes of neurovascular coupling, which can be studied experimentally or by using complex mathematical modeling [8,9,10].

It was found that epileptic seizures induce long-term increases rather than decreases in local cerebral oxygenation as well as increases in local blood circulation and is a reliable marker of an underlying epileptic discharge [11]. Massive firing of neurons in the epileptic foci increases energy consumption by local brain cells. This energy is produced by cellular metabolism from oxygen and glucose supplied by blood through the capillary network. Thus, in response to transient local seizures, nearby capillaries need to increase local blood circulation. This mechanism, known as neurovascular coupling, is defined as the physiological linkage between transient neural activity and the regulation of cerebral blood circulation, and this is common during normal functioning of the brain [12,13]. Functional magnetic resonance imaging (fMRI), intrinsic optical imaging (IOS), and near-infrared spectroscopy (NIRS) can measure blood oxygenation variations associated with transient neural activity [14,15,16]. Optical methods in animal models are currently used to measure blood circulation intensity, flow changes, and local oxygenation in the cerebral cortex and are routinely interpreted as changes in neuronal activity [10,17,18,19,20,21,22]. However, the relationship between epileptic seizures and local neurovascular coupling processes is much more complex. Despite the numerous findings obtained in recent years, the potential mechanisms underlying the relationship between changes in local blood vessels and pathologically synchronized neurons remain unclear.

Using modern brain imaging methods, it was previously demonstrated that increases in cerebral blood flow (CBF) occur after the onset of epileptic seizures [23,24]. It was also shown that the metabolic rate of oxygen consumption and CBF have no direct relationship during an ictal event [23]. Another study reported a fast decrease in local oxygenation that preceded the increase in CBF in the seizure area. This phenomenon, known as the “initial dip”, although controversial, proves that, for a brief period of time after ictal onset, neurons experience oxygen deficiency until cerebrovascular regulation dilates vessels to augment blood circulation [22].

It should be noted that the relationship between vasodilation and an ictal event is perhaps not so simple. It cannot be stated unequivocally that seizure onset begins completely independently of local vasodilation and other changes in neurovascular coupling [22]. Moreover, the reactions of blood vessels and the adjacent astrocytic syncytium possibly contribute to triggering and supporting epileptic seizures, at least in some cases [11,22].

In this study, we hypothesized that cerebral autoregulation may be impaired in the zone of formation of epileptic seizures. As is well known, tonic–clonic seizure-like events can be induced by elevation of K^+^ or lowering of Ca^2+^ or Mg^2+^ in the extracellular space [22,25]. As a non-selective potassium channel blocker, 4-amynopiridine (4-AP) inhibits K^+^ outward current, which, in turn, causes prolongation of action potentials and increases the excitability of both inhibitory and excitatory neurons, the former of which is pivotal in the development of epileptic seizures. The 4-AP model generates epileptic seizures lasting from a few tens up to a few hundreds of seconds, with periods between seizures lasting minutes [4,10].

Our task was to study the reactions of neurons, blood vessels, and astrocytes in the area of epileptic activity. We performed experimental studies both in vitro and in vivo, which allowed us to obtain uniquely comprehensive results. Although in vitro experiments on living brain slices using the 4-AP model are common [26], we used slices containing a fragment of a pressurized blood vessel that is rarely used because it need special experimental dexterity. Unlike the simulation of the blood flow with chemical preconstruction, that might evoke a myogen response, this experimental method allowing more natural conditions for blood vessels during the experiment [27,28].

The presence of a small fragment of blood vessel in a living slice of brain tissue allows not only investigation of neuronal activity by electrophysiological and optical methods but also monitoring of the reactions of pressurized blood vessels during the seizure. Combined use of the 4-AP model of epileptic seizures in vitro and in vivo has provided interesting results that may shed light on neurovascular coupling in the epileptic seizure area.

## 2. Materials and Methods

In total, 18 adult rats (male, 250–400 g, 3–5 months) were used for in slice as well as in vivo experiments. Ten male Wistar rats, which was originally obtained from Animal Resource Center, Universidad Central del Caribe (Bayamon, Puerto Rico) and maintained in Universidad Central del Caribe animal facilities, were used for in vitro experiments. Eight male Wistar rats used for in vivo experiments were obtained from Rappolovo Nursery, Russian Academy of Medical Sciences, (St. Petersburg, Russia) and maintained in the Saint Petersburg University animal facility. All procedures involving rodents were conducted in accordance with the National Institutes of Health (NIH) regulations concerning the use and care of experimental animals and approved by the UCC Institutional Animal Care and Use Committee (IACUC, for in vitro experiments, approval #10-XI-00) and the Ethical Committee for Animal Research of Saint Petersburg State University (for in vivo experiments, approval #131-03-4). Surgical procedures were performed using sterile/aseptic techniques in accordance with institutional and NIH guidelines. To minimize discomfort, the animals were anesthetized in all procedures involving surgery and before euthanasia.

### 2.1. In Vitro Study of Vasomotor Responses

#### 2.1.1. Brain Slice Preparation and Patch-Clamp

**Figure 1:**
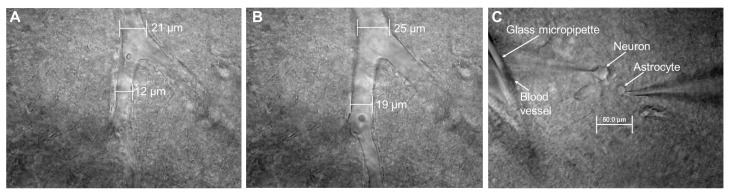
Perfusion of the blood vessel in a brain slice with artificial cerebrospinal fluid (ACSF). Photomicrograph of a blood vessel in the living brain slice under normal conditions (**A**) and when pressure is applied inside the vessel (**B**). (**C**) Blood vessel with microcapillary inserted inside and the neuron and astrocyte with attached microelectrodes. A glass electrode was inserted inside the blood vessel in a 400 μm brain slice preparation, and 20–30 torr pressure (see text) was applied.

In total, 10 rats between 30 and 60 days of age were rapidly decapitated. Hippocampal slices (400 μM) were prepared using a vibratome (VT1000S, Leica Microsystems GmbH, Wetzlar, Germany) in artificial cerebrospinal fluid (ACSF) containing (in mM) 127 NaCl, 2.5 KCl, 1.25 NaH_2_PO_4_, 25 NaHCO_3_, 2 CaCl_2_, 1 MgCl_2_, and 25 d-glucose, ice cold, saturated with a 95% O_2_/5% CO_2_ gas mixture at pH 7.4. Totally 16 slices from 10 different animals were used. Slices were perfused (0.1 mL/sec) with the same ACSF at room temperature. For whole-cell recordings, membrane currents and voltages were measured with the single-electrode patch-clamp technique. Cells were visualized using an Olympus infrared microscope fixed on an X-Y stage (Narishige Int. Group, Japan) and equipped with differential interference contrast (model BX51WI, Olympus, Japan). Two piezoelectric micromanipulators (MX7500 with MC-1000 drive, Siskiyou, Inc., Grants Pass, OR, USA) were used for voltage-clamp and current-clamp recording. An additional two MN4 manipulators (Narishige Int. Group, Japan) were used for a local field potential (LFP) electrode and a pressurizing micropipette. All manipulators and microscopes were separately fixed to an anti-vibration table (VH-AM, Newport Corporation, CA, USA). A MultiClamp 700A patch-clamp amplifier with a DigiData 1322A interface (Molecular Devices, Inc., Sunnyvale, CA, USA) was used for recording and stimulation. The pClamp-10 software package (Molecular Devices, Inc., CA, USA) was used for data acquisition and analysis. Borosilicate glass pipettes (O.D., 1.5 mm; I.D., 1.0 mm; World Precision Instruments, Sarasota, FL, USA) were pulled to a final resistance of 8–10 MΩ for astrocyte recordings in four steps using a P-97 puller (Sutter Instrument Co., Novato, CA, USA). Electrodes were filled with the following solution (in mM): 130 K-gluconate, 10 Na-gluconate, 4 NaCl, 4 phosphocreatine, 0.3 GTP-Na_2_, 4 Mg-ATP, and 10 HEPES, and the pH was adjusted to 7.2 with KOH. Astrocyte recordings were considered only if the membrane potential (MP) was negative, up to −80 mV, and there was low input resistance (<20 MΩ). Experiments with a brain section and electrodes were performed using an infrared video monitoring system (Figure 1C). Constant video monitoring allowed us to control that there are no movements of the slice as a whole during ictal events or large swelling occurs during the experiments.

#### 2.1.2. To Pressurize Blood Vessels in the Slice

A glass electrode with a tip of ~20 µm in diameter was filled with ACSF and fixed in a standard patch-clamp holder, while the holder was connected to a pressure control and management system. We used relatively large blood vessels, with a diameter of 100–150 µm, lying mainly in the plane of the slice. We picked the cut end of the vessel visible on the slice surface. The slice was oriented so that the tip of the glass electrode rested against the cut end of the vessel. The tip of the pressure electrode was then moved inside the vessel using a micromanipulator. Next, a pressure of 0–50 torr was applied. The smaller derivative vessels of 10–20 µm in diameter were inspected to see whether these vessels changed in diameter at different pressures, and the limits of vessel diameter were determined (maximum diameter, minimal diameter) (Figure 1A,B). A mean pressure of 20–30 torr was sustained continuously throughout the experiment to maintain the diameter in the middle of the diameter range thus providing the vessel a dilation ability.

Piezo-electrode: To make a mechanosensitive electrode, borosilicate glass electrodes were pulled so that the tip of the electrode became ~1 µm (for intracellular recording) and then additionally heated in a micro-forge for 10 min. After extensive heating, the electrode became mechanosensitive and generated a piezo potential of ~0.1 mV/µm upon tip bending, due to ceramic formation [29]. This electrode was then installed in a micromanipulator with a standard patch-clamp holder, connected to an isolated voltage amplifier (DP-301, Warner instruments, Holliston, MA, USA), positioned against the vessel wall, and calibrated using a microscope to monitor vasomotor activity. The second channel of the amplifier was used to connect a standard low-resistance electrode to record the LFP (filtered at 0.1 Hz with a high-pass filter) from the slice.

Two glass microcapillaries were used for simultaneous patch-clamp recording from a closely neighboring neuron and astrocyte or two astrocytes, also simultaneous with LFP activity and vasomotor activity in our experiments.

### 2.2. In Vivo Imaging of the Epileptic Seizures

For each in vivo experiment 8 Wistar adult rat were anesthetized with an i.p. injection of a mixture of ketamine (90 mg/kg) and xylazine (12 mg/kg) and fixed onto a stereotaxic frame. On the dorsolateral part of the skull, a cranial window of ∼5–7 mm^2^ was made. After craniotomy, intracortical injection of 0.3 µL of a 25 mM solution of 4-AP in artificial cerebrospinal fluid (ACSF) was done by Hamilton syringe injection at 0.5 mm below the surface to induce local epileptic seizures. To suppress cortical tissue motion induced by breathing and heart rhythm, the region of interest was covered with mineral oil and a cover glass. For LFP recording, a metal high-impedance microelectrode (glass-coated tungsten, R ≅ 1 MΩ) was positioned at the region of interest at about 0.5 µm below the cortical surface. The reference electrode was a silver plate (1–2 mm^2^) implanted over the cerebellum. Neural activities were fed into a multichannel amplifier (USF-8; Beta Telecom), band-pass filtered at 0.1–200 Hz, and digitized. A 12-bit CCD camera (QuickCam, 640 × 480 pixels) with a built-in objective was focused 0.5 µm below the cortical surface around the 4-AP injection site, and images were acquired with at 3 fps. We used an algorithm comparing frames obtained in the ictal and in the interictal periods, and corresponding frames were selected during off-line analysis based on LFP data.

Illumination was provided with an LED at 630 nm (red light), which was homogeneously projected onto the region of interest. All images were analyzed off-line. At each time point, the averaged light reflection intensity of the pixels within the region of interest was quantified and normalized to the mean baseline value. Pseudocolor images were obtained by digitally amplifying the difference with a zero-image using the Metamorph program (Universal Imaging Corp., Downingtown, PA, USA) and the histological Photoshop plugin. We compared the width of vessels during spike-wave discharges (SWD) to the width of the vessel in the interictal period, which was taken as 100%.

### 2.3. Chemicals and Materials

All chemicals and materials not specially mentioned were purchased from Sigma-Aldrich (St. Louis, MO, USA).

### 2.4. Statistics and Measurements

GraphPad Prism 7.03 (GraphPad Software, Inc., La Jolla, CA, USA) was used for calculations of the Kolmogorov–Smirnov normality test, the ordinary *t*-test and one-way ANOVA to determine statistical differences, as indicated for each experiment. Values were determined to be significantly different if the *p*-value was <0.05.

## 3. Results

### 3.1. In Vitro Experiments

**Figure 2:**
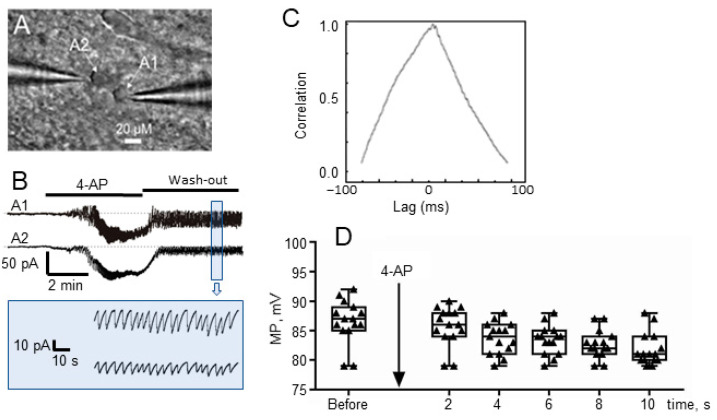
(**A**) Two astrocytes, A1 and A2, with microelectrodes attached. (**B**) Recording of the astrocyte currents. Simultaneous high-amplitude inward currents and high-frequency activity in the astrocytes shown in (**A**) after application of 4-AP and washout. (**C**) Cross correlation function (CCF) graph of recordings in (**B**) with a value of 0.98 at lag time = 0. (**D**) Effects of 4-AP application on astrocytes MP, before and during 10 min after 4-AP application (data shown are mean M ± standard error of mean, SEM, and min–max).

The potassium channel blocker 4-AP (100 µM) in ACSF perfusion solution was applied to the hippocampal slice, totally 16 slices from 10 different animals were used. Patch-clamp was performed on astrocytes and neurons near a pressurized vessel using voltage-clamp and current-clamp modes, respectively. Patch-clamp of a pair of nearby astrocytes in voltage-clamp mode (Figure 2A) showed that a powerful inward current (corresponding to temporary astrocyte depolarization) in the astrocyte membrane occurred simultaneously with the onset of seizure-like events and usually started 2–5 min after the application of 4-AP (Figure 2B). These depolarization events occurred randomly throughout the recording without a regular period, and were observed in all astrocytes that we were able to study. Upon washout, these depolarization events, which at first lasted a few minutes, became shorter over time after 4-AP application. They then shortened to 10–20 sec duration and finally disappeared after 30–40 min after application of 4-AP.

It was found that membrane potential (MP) of astrocytes was significantly reduced (Figure 2D) from −86.5 ± 1.0 mV to −82.0 ± 0.8 mV about 10 min after the application of 4-AP (paired *t*-test: *p* = 0.0017, t = 3.9, df = 14, *n* = 15). The one-way ANOVA post-test for linear slope showed that there is a statistically significant linear trend for MP during 10 min (*F*(1, 70) = 76.9, *p* < 0.0001).

Patch-clamp electrodes, which record the astrocyte MP, also recorded relatively high-frequency, low-amplitude spike-like oscillations, corresponding in shape and frequency to the known electrical activity of epileptic seizure-like event (*M* = 0.43 Hz, *SD* = 0.06). The duration of this seizure-like activity was 0.5–3 min (average, 75 s), and these events were recorded both by the extracellular electrode and by patch-clamp of the nearest astrocytes. As well as powerful depolarization of the astrocyte membrane, high-frequency, low-amplitude oscillations caused by 4-AP were recorded on all astrocytes during epileptic seizure periods. These oscillations started almost simultaneously with, and persisted along with, a deep inward-current (depolarization, Figure 2B) and had not ended after 30–40 min of 4-AP washout. Activity in the nearby astrocyte pair occurred synchronously (Figure 2B), with a very clear positive correlation, Pearson correlation coefficient was 0.97. Analysis of cross-correlation lag values showed a zero-lag time (+0.98 at lag time = 0. calculated with standard Clampfit V10.2-012 cross-correlation function, Figure 2C). Thus, we clearly observed that nearby astrocytes generated their low-amplitude, high-frequency discharges with a high degree of synchronization.

**Figure 3:**
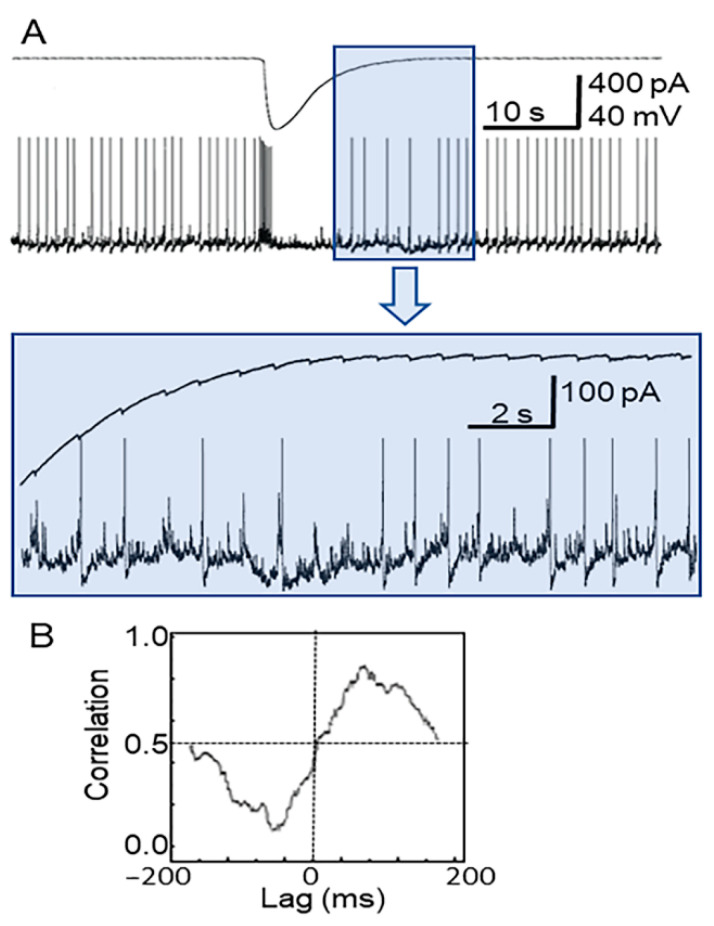
(**A**) Simultaneous patch-clamp current recordings of an astrocyte (upper trace, pA) and a neuron (lower trace, mV) corresponding to the seizure-like event elicited by 4-AP. The colored insert (above) shows the same recording at greater time and amplitude resolution. (**B**) Cross correlation function (CCF) graph of recordings in (**A**) with a value of +0.88 at lag time = 100 ms.

Simultaneous recording from neurons (CA1 zone, hippocampus) and nearby astrocytes enabled an understanding of how their activities after 4-AP application are related (Figure 3A, upper and lower traces). The upper trace is for an astrocyte recorded in voltage-clamp mode, while the lower trace is for the neuron (current-clamp mode). As in previous experiments, 100 µM 4-AP in ACSF perfusion solution was applied to the hippocampal slice over a period of 4 min with a following washout [30]. This led to seizure-like activity in the slice, corresponding to occasional bursts of synchronized spike activity in neurons (Figure 3A, lower trace). Simultaneous recordings show that the neuronal bursts corresponded to a high-amplitude, 15 s inward current (corresponding to temporary depolarization) in the astrocyte (Figure 3A, upper trace). After this event, fast spike-like oscillations appeared in the astrocytes, which corresponded with spikes or giant EPSPs in the neuron (Figure 3A, colored insert). The cross correlation function between neuronal activity and the high-frequency, low-amplitude activity of astrocytes (Figure 3B) was also high (CCF = +0.88, Clampfit, cross-correlation), with a stable delay different for each neuron–astrocyte pair, which can be described as a constant phase shift.

**Figure 4:**
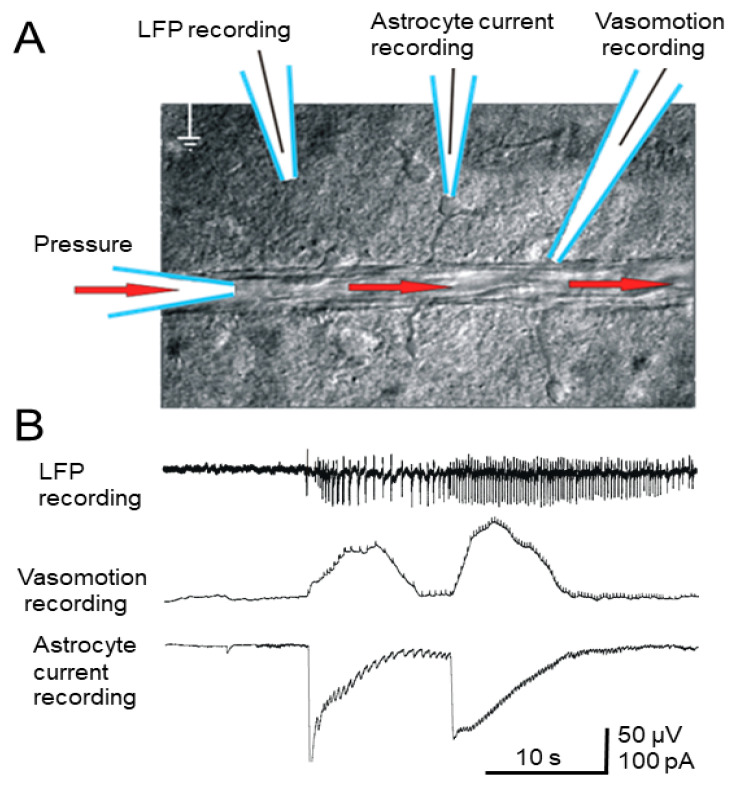
(**A**) Positioning of the blood vessel perfused with ACSF (30 torr) and recording electrodes. (**B**) Simultaneous extracellular LFP, vasomotor, and astrocyte patch-clamp recording after 4-AP application (see text). Low-voltage, high-frequency components superimposed on the vasomotor response, but did not reflect actual vasodilation, reflecting neural spikes.

A blood vessel (diameter 100–150 μm) was perfused with ACSF at constant pressure through the inserted micropipette, while smaller derivative blood vessels were also pressurized. The electrode positioning is shown (Figure 4A). A mechanosensor was positioned on the pressurized blood vessel, and movements of the blood vessel wall were recorded during the seizure, which corresponded to large inward currents in the astrocytes (Figure 4B). Application of 4-AP caused epileptic activity in the slice, and this activity was clearly visible by the LFP as a high-frequency, high-amplitude event (Figure 4B). The mechanosensor recorded a distinct vasoconstriction response, accompanied by electrophysiological correlates. The effect of vasoconstriction caused by seizure-like event corresponded only to slow inward currents (producing temporary depolarization) in astrocytes, but later high-frequency spike-like oscillations in astrocytes corresponding to seizure-like events (according to the LFP) were also observed (Figure 4B). This effect was stable and was observed in all cases when seizure-like activity occurred simultaneously with the application of pressure inside the vessel. No vasomotor activity was observed between seizure-like events, regardless of whether pressure was applied inside the vessel. We also failed to find correlations between the intensity of vasoconstriction and the pressure applied to the vessel. Vasoconstriction started at the same time as the epileptic seizure and ended when a long-lasting inward current (depolarization) event in the astrocyte ended. Seizure-like event recorded according to the LFP in most cases began with a single high-amplitude discharge, and closer to the end of the recording the amplitude of the spikes decreased.

Vasomotor reactions detected by the mechanosensor are inextricably linked to changes in neuronal activity as well as changes in inward currents in the astrocytes in the epileptic seizure zone (Figure 4B).

### 3.2. In Vivo Experiments

In vivo experiments were performed on the whole brain using intrinsic optical imaging in combination with LFP recording. Seizure-induced optical responses were monitored by IOS imaging within the cortical region. Intracortical injections of 4-AP induced recurrent seizures, typically lasting a few seconds for each event (17.6 ± 3.8; from 6.9 to 32.5 s) and with events recurring for about 2 h after the injection [31,32].

**Figure 5:**
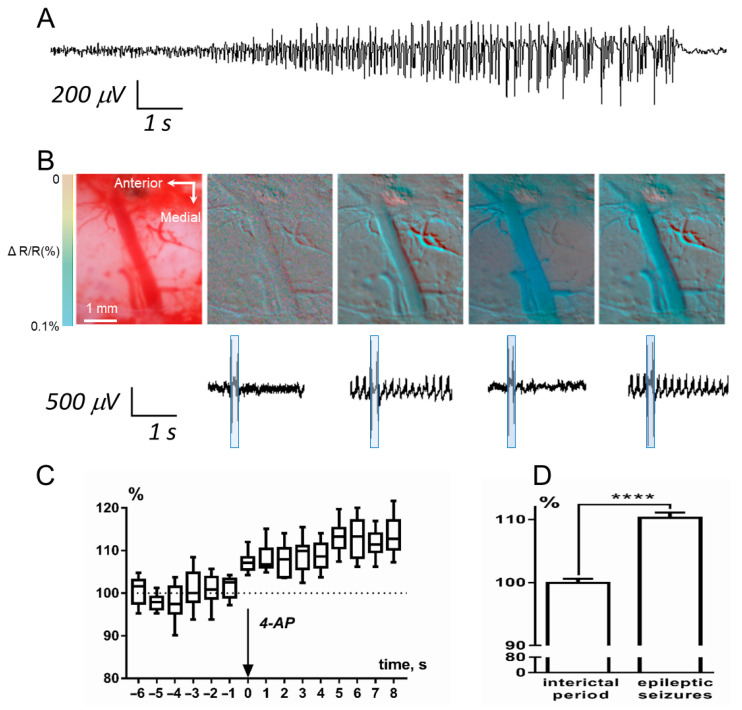
Simultaneous recording of LFP and in vivo visualization of epileptic seizures using the IOS imaging. (**A**) LFP epileptic seizure. (**B**) Pseudocolor images of the zone where epileptic seizures were induced. The first image on the left is a color image of a region of the cortex. The following images are pseudocolor images corresponding to the LFP-recording fragments shown below. The pseudocolor boxes superimposed on the LFP-recording fragments indicate the moment of capture of the frame indicated by the arrow. (**C**) Diagram of diameter of blood vessels changes showed statistically significant linear trend there was only after 4-AP injection (box-and-whiskers diagram with min and max). (**D**) The changes in the lumen of vessels outside and during SWD; results are presented as mean ± SEM, **** *p* < 0.0001 (Student’s *t*-test).

A seizure was defined as a series of discrete spikes, with an onset consisting of high-frequency discharges (10.59 ± 0.21 Hz), followed by an evolving rhythmic, high-amplitude activity (3.04 ± 0.05 Hz) with a distinct termination (Figure 5A). In most cases, epileptic seizures began with a single high-amplitude spike, and the frequency of spikes increased over the course of the seizure. During the IOS session, we recorded an optical signal from the region of interest, with a frame duration of 0.1 s and a frequency of 1 frame every 10 s. The beginning and end of each frame were marked on the LFP recording with specific double-spike artifacts (Figure 5B). In all cases, decreased light reflectance was observed in the epileptic foci during the epileptic seizures compared with the interictal period. Data were collected before, during, and for up to two hours after epileptic seizure induction, and acquired images were stored digitally. In all experiments, IOS changes showed statistically significant linear trend only after 4-AP injection (*F*(1, 81) = 31.9, *p* < 0.0001) (Figure 5C), before injection there was no statistically difference in this parameter.

As is well known, in the part of the spectrum that we used (~630 nm), a decrease in reflection is associated with an increase in hematocrit. In the case of relatively large blood vessels, an increase in hematocrit is almost exclusively associated with vasodilation. Through comparison of the simultaneously acquired LFP and IOS data, we observed vasodilation corresponding to epileptic seizures (Figure 5D). We analyzed changes in the lumen of vessels outside and during spike-wave discharges (SWD), the width of the vessel in the interictal period was taken as 100%. Vasodilation during a seizure was significant, it was on average 110.3 ± 0.5% (one-sample *t*-test, H0: mean equals 100%; *n* = 90 t =23.3 df = 89; *p* < 0.0001).

These hemodynamic changes confirmed an increase in local microcirculation before the seizures, and the elapsed time between vasodilation and seizure onset depended on the distance from the site of the 4-AP injection. Without exception, in all experiments there was a significant increase in the diameter of blood vessels, correlating with the duration of epileptic seizures. The onset and termination of this vasodilation were delayed by electrophysiologically recorded epileptic seizures for no more than 1 s. The vessel appeared in a dilated state for the entire duration of the epileptic seizures and narrowed simultaneously with the end of the seizures. As recorded with the LFP electrode, pre-seizure activity was relatively low, and strong IOS was observed only during electrographic seizures.

## 4. Discussion

Epileptic seizures are a complex phenomenon that, with different pathways of development, can trigger both vasoconstriction and vasodilation. Vasodilation and vasoconstriction caused by epileptic seizures have been repeatedly described in the literature. Using different methodological approaches, it was shown that epileptic seizures are accompanied by local vasodilation, and this phenomenon is associated not only with pathological synchronization of neurons but also with slow depolarization of the astrocyte membrane [33]. It should be noted that the same authors showed that electroconvulsive seizures caused a rapid elevation in astrocyte endfoot Ca^2+^ that was confined to the seizure period. Vascular smooth muscle cells expressed a significant elevation in Ca^2+^ both during and following seizures. [34]. Additionally, they found biphasic reaction: arterioles dilated in response to the seizure, with a decreasing amount of dilation with increasing distance from the 4-AP injection site. The biphasic reaction (vasoconstriction in the preictal period and vasodilation during a later stage of the seizure) was only evident in the remote area [35].

Thus, increases in Ca^2+^ in astrocyte endfeet correlating with vasoconstriction at the onset of seizure and with vasodilation during the latter part of the seizure have been shown [36]. It was also shown that pericytes are involved in the control of capillaries’ vasomotion and depolarization of pericytes in the postictal phase can lead to vasoconstriction [12,37].

Usually, epileptic activity initiates a local increase in cerebral metabolism and CBF, but decreases in CBF have been demonstrated surrounding the epileptic focal area [35]. An imbalance of inhibition and excitation causes neural network hyperexcitability and eventually leads to seizures. The network activity of neural and glial cells is an important factor that regulates the multidimensional response of the vascular system, including the interaction between interconnected blood vessels [2]. Early epileptic studies suggested that vasospasms caused by seizures led to local ischemia, but later hyperemia—the opposite of ischemia—was found in the epileptic seizure area [38,39]. Seizures induce reversible vasodilation and increases in the local blood flow, resulting in an overshooting supply of oxyhemoglobin [40]. Using several models of epileptic mice, it was demonstrated that vasospasms are more likely to occur in the ictal zone capillaries of epileptic mice than in control animals [40].

The 4-AP model of epileptic seizures allows us to investigate pathologically synchronized neural activity in vitro as well as in vivo [41]. We used a unique technique of applying artificial pressure to the interior of a fragment of a vessel located in a living brain slice. This technique allows one to simultaneously register vasomotor activity and the cellular activity of neurons and astrocytes. In our experiments we found that 4-AP induced synchronized neural activity led to significant potassium release (Figure 3, lower trace). Simultaneously, there was an MP change in astrocytes due to a high-intensity inward current, as the astrocytes tried to absorb the potassium (Figure 3, upper trace), with the phase probably corresponding to the beginning of the ictal period. These synchronized neurons then produced simultaneous spikes, reflected as “high-frequency” signals in astrocytes (Figure 2 and Figure 3). Each individual neuronal spike corresponded to a fast spike-like oscillation in the astrocyte with a stable delay, as is illustrated in Figure 3. All astrocytes near these synchronized neurons had their high-frequency signals synchronized with zero lag time (Figure 2).

If we compare LFP signal recorded by an extracellular electrode in vitro and filtered through a high-pass filter (Figure 4), we can see that it resembles an EEG signal recorded during in vivo studies (Figure 5) within the ictal period (Figure 5A). By contrast, high-diameter arteries in in vivo experiments were clearly dilated during the beginning of the ictal period (Figure 5C,D), while our in vitro experiments on brain slices showed that there is clear vasoconstriction in small diameter arterioles accompanying astrocyte activity (Figure 4). Our data obtained in vivo using a 4-AP model of epileptic seizures corresponds with other reports of ictal events rapidly accompanied by local vasodilation of relatively large (≥50 µm) vessels in the zone of epileptic seizure [36]. Meanwhile, optical methods have shown that low oxygenation (probably corresponding to vasoconstriction) is recorded in the focal location of the ictal zone, while in the peripheral blood vessels the CBF becomes reduced [42].

The results obtained in our study that relate astrocyte membrane currents to vasoconstriction during seizures are consistent with other studies obtained in recent years with epileptic models [35]. Normally, astrocytes are able to remove a large amount of K^+^ from the extracellular space, since it can be spatially buffered via redistribution through gap junctions into the syncytial network of gap junction-coupled astrocytes [43,44]. Therefore, any pathological change in gap junction coupling could impact astrocytic functions and may contribute to seizure occurrence. In the case of 4-AP models, gap junctions function normally, but extracellular K^+^ rises due to neuronal hyperactivity.

Our results demonstrate that in a model of in vitro epileptic seizure, event onset is accompanied by vasoconstriction in small blood vessels, while during the seizure large vessels exhibit vasodilation, as was found previously [35]. Three-dimensional seizure events are complicated, and the phenomenon of vasoconstriction in the epileptic foci have been challenging to study in vitro. It might be why it has been suggested that various forms of epileptic activity only increase local cerebral blood circulation. For example, it was demonstrated using the bicuculline seizure model that CBF increases dramatically with seizure onset, reaching a maximum after 15–60 s [45].

In summary, mechanosensor recordings allowed us to describe properties of synchronous epileptiform discharges and vasomotor activity induced by 4-AP in an in vitro living cortico-hippocampal slice. We observed that neuronal oscillations during an epileptic seizure precede fast spike-like events in astrocytes. Since astrocytes are combined into an astrocytic syncytium by gap junctions, they start to remove potassium from the extracellular space locally, but the resulting current spreads throughout the syncytium. This, in turn, supports the existence of pathological neural depolarization. This process is accompanied by local vasomotor activity, which is closely related to neuronal and astrocytic activity, but its biological role is not entirely clear.

## 5. Conclusions

Our results obtained in vitro and in vivo reveal a relationship between ictal events in neurons and astrocytes and vasomotor events. There remains little doubt that astrocyte properties contribute to epileptic seizure onset, spread, and termination, which can be attributed to their synchronous depolarization that follows neuronal oscillations. Moreover, using a combination of in vitro vasomotor activity recordings and IOS imaging in vivo, we observed local constriction of small blood vessels and dilation of relatively large blood vessels at different time points before and during the seizure.
